# Bovine Tuberculosis and Brucellosis in Traditionally Managed Livestock in Selected Districts of Southern Province of Zambia

**DOI:** 10.1155/2013/730367

**Published:** 2013-06-13

**Authors:** J. B. Muma, M. Syakalima, M. Munyeme, V. C. Zulu, M. Simuunza, M. Kurata

**Affiliations:** ^1^Department of Disease Control, School of Veterinary Medicine, University of Zambia, P.O. Box 32379, Lusaka, Zambia; ^2^Dale Beighle Centre for Animal Health Studies, Faculty of Agriculture, Science and Technology, Private Bag X2046, Mmabatho, South Africa; ^3^Department of Clinical Studies, School of Veterinary Medicine, University of Zambia, P.O. Box 32379, Lusaka, Zambia; ^4^Amimal Disease Diagnosis Project, School of Veterinary Medicine, Makerere University, P.O. Box 12162, Kampala, Uganda

## Abstract

A study was performed in 2008 to estimate the prevalence of tuberculosis and brucellosis in traditionally reared cattle of Southern Province in Zambia in four districts. The single comparative intradermal tuberculin test (SCITT) was used to identify TB reactors, and the Rose Bengal test (RBT), followed by confirmation with competitive enzyme-linked immunosorbent assay (c-ELISA), was used to test for brucellosis. A total of 459 animals were tested for tuberculosis and 395 for brucellosis. The overall prevalence of BTB based on the 4 mm and 3 mm cutoff criteria was 4.8% (95% CI: 2.6–7.0%) and 6.3% (95% CI: 3.8–8.8%), respectively. Change in skin thickness on SCITT was influenced by initial skin-fold thickness at the inoculation site, where animals with thinner skin had a tendency to give a larger tuberculin response. Brucellosis seroprevalence was estimated at 20.7% (95% CI: 17.0–24.4%). Comparison between results from RBT and c-ELISA showed good agreement (84.1%) and revealed subjectivity in RBT test results. Differences in brucellosis and tuberculosis prevalence across districts were attributed to type of husbandry practices and ecological factors. High prevalence of tuberculosis and brucellosis suggests that control programmes are necessary for improved cattle productivity and reduced public health risk.

## 1. Introduction

Bovine tuberculosis and brucellosis are major zoonotic diseases of worldwide economic and public health importance, especially in developing countries where the diseases are endemic [1]. In some developed countries, these diseases have been brought under control, with subsequent benefit to public health and decrease in associated economic losses. In developing countries, the public health importance of these zoonoses is often overshadowed by the “big three” diseases, malaria, HIV/AIDS, and human tuberculosis caused by *Mycobacterium tuberculosis *[1]. Thus, diseases such as bovine tuberculosis and brucellosis, which are often associated with resource poor communities, are now termed “neglected zoonoses” probably as a way of raising awareness that something needs to be done to give these diseases their deserved attention [1].

*Mycobacterium bovis* is a member of *Mycobacterium tuberculosis* complex, which also includes *M. tuberculosis, M. africanum, M. microti, M. caprae,* and *M. pinnipedii* [2]. Infections in animals are often subclinical, but when present, clinical signs may include weakness, dyspnoea, enlarged lymph nodes, coughing, and extreme emaciation, particularly in advanced cases. Bovine tuberculosis is usually diagnosed using the delayed hypersensitivity reaction although culture still remains the gold standard [2]. In a study conducted in Mexico, Milián-Suazo et al. [3] observed that out of the 562 human samples obtained from TB-symptomatic cases, analysed for detection of mycobacterial infections, 34 (6%) showed *M. bovis* spoligotype and concluded that infected cattle presented a risk to public health. In another study conducted in Tanzania, *M. bovis* was isolated from 7 out of 65 (10.8%) human cases of cervical adenitis in HIV-infected person [4, 5].

Human brucellosis is one of the widely distributed zoonoses, especially in economically disadvantaged livestock keeping communities [1]. In humans, brucellosis is typically caused by four *Brucella* species: *Brucella melitensis, B. abortus, B. suis,* and *B. canis* [6]. Of these, infections by *B. melitensis* are reportedly more severe, and the agent remains the principal cause of human brucellosis [7], often transmitted from sheep or goats [8, 9]. The disease is mainly transmitted to humans through ingestion of raw milk or nonpasteurized cheese [10]. Human exposure also occurs through contact with infected livestock, particularly when they are aborting. Infection route may be the respiratory tract, conjunctiva, or broken skin [11]. Diagnosis of brucellosis is mainly through serological tests since they are easy to perform [12], but like tuberculosis, culture is the gold standard for definitive *Brucella* diagnosis. There are few reports on human brucellosis in sub-Saharan Africa, but it is assumed that many cases go undetected [13, 14]. Infections with *B. melitensis* and *B. abortus* in South Africa, Sudan, and Tanzania have been reported [15–17]. 

In the light of high HIV/AIDS prevalence in sub-Saharan Africa [18–21], animal protein (meat and milk) is highly required to mitigate the impact of the HIV/AIDS pandemic, but cattle infertility resulting from *Brucella* infections is likely to reduce milk yield [22]. BTB and brucellosis in cattle might lead to reduced productivity, increased risk of abortion, and lowered calving rates resulting in decreased milk production [23–25]. In Zambia, BTB has been shown to be one of the major leading causes of carcass condemnations in some abattoirs while Brucella infections account for high proportion of cattle abortions [26]. 

Although the livestock keeping communities are at highest risk of contracting these zoonotic diseases, they are often unaware of these risks. In our earlier reports, we noted that there is generally low awareness among the traditional farmers regarding the risk to *M. bovis* [27] and *Brucella spp.* infections [28]. This study was undertaken as part of a wider project to improve veterinary extension and delivery of veterinary service under the auspices of the Japan International Agency (JICA) and the Ministry of Agriculture and Cooperatives (MACO). Therefore, the aim of this study was to investigate the disease status of BTB and brucellosis in Southern Province (project areas). 

## 2. Materials and Methods

### 2.1. Study Areas

The study was conducted in four districts of Southern Province in 2008. These districts were purposively selected because these were the operational areas of the funding project and also because most cattle under traditional management are found in these areas (Table 1). Cattle found in these areas were mainly the Zebu and Sanga breeds with a small fraction of mixed breeds. Cattle in the study areas were typically grazed communally on land held in trust by local chiefs. Some farmers practice transhumant grazing, defined as seasonal migration of livestock to suitable grazing and watering areas. In this case, animals are moved to the flood plains of the Kafue river immediately after the harvest season (from March to May) and returned to the upland with the onset of rains (from November to December). Some herds are grazed permanently in the flood plains of the Kafue River.

### 2.2. Study Design

The study was conducted as a cross-sectional study to estimate prevalence of antibodies to *Brucella *and reactivity to bovine tuberculosis (BTB). Only animals aged ≥2 and above were included in the study. The cattle population was divided into strata based on the districts (Table 2). A multistage sampling strategy was adopted for each district with veterinary camps as primary and herds as secondary sampling units and unit of interest. Sampling of veterinary camps was based on the list obtained from the District Veterinary Office while sampling of herds was based on lists of farmers generated with the help of local veterinary paraprofessionals (where available) and some farmers. Herds reared in close proximity were considered as one, and only herds with ≥10 animals were included in the study.

### 2.3. Sample Size Calculation

We assumed that sampling would be done randomly and that there would be low heterogeneity between herds. The detection power was set (1 − *β*) at 90% and level of significance (*α*) = 5% at an estimated herd prevalence of 20% for both BTB and brucellosis. Based on these assumptions, the number of cattle herds and individuals to be sampled were estimated (Table 2) using the simple random formula as indicated by Dohoo et al. [29]. 

### 2.4. Intradermal Skin Test for BTB Diagnosis

For the determination of the prevalence of BTB in cattle, the single comparative intradermal tuberculin test (SCITT) was applied. The procedure was conducted as earlier described [2]. Briefly, two circular areas of about 2 cm in diameter were clipped in the cervical region, washed with soap, and disinfected with alcohol. The initial reading in skin thickness (preinjection skinfold thickness) was measured using a caliper followed by inoculation of 0.2 mL, intradermally at each respective site, of bovine and avian purified protein derivatives (PPD) manufactured by ID Lelystad, the Netherlands. The result of hypersensitization was read and recorded in millimeters 72 hours after injection by measuring again the skinfold thickness. Interpretation of results was done as earlier prescribed [30]. 

### 2.5. Serum Samples for Brucellosis Diagnosis

Blood samples were collected from pregnant heifers, cows, and bulls, since clinical brucellosis is said to be a disease of sexually mature animals (≥2 years) [31]. Sampling of animals was done randomly. Blood samples were clotted at room temperature. 

### 2.6. Laboratory Analysis

In the laboratory, sera was separated by centrifugation at 2,500 rpm (503 g) for 15 minutes and stored in 2 mL cryovials at −20°C until laboratory tests were performed. Antibodies to *Brucella *spp. were detected in sequential testing of samples using Rose Bengal test (RBT) for screening and c-ELISA for confirmation. RBT was done as described by [31]. Details of the test procedures are described elsewhere [32]. In order to improve the results of the RBT, we engaged two technicians who independently tested the same set of sera. Technician 1 (RBT-1) was elderly and had aided sight while technician 2 (RBT-2) was young and had no aided sight.

### 2.7. Data Analysis

 The database was established in Excel before transferring to STATA SE 11 for Windows (StataCorp, College Station, TX).

#### 2.7.1. Tuberculosis

BTB-positive reactors (mm) and avian-positive reactors (mm) were obtained as earlier described [33], using the following formulae: ((Bov72 − Bov0) − (Av72 − Av0)) and ((Av72 − Av0) − (Bov72 − Bov0)), respectively. Bov0 and Av0 indicated skin thicknesses before injecting bovine and avian tuberculins, respectively, and Bov72 and Av72 were the corresponding skinfold thicknesses 72 h after injection. Two criteria were used for assessing reaction to SCITT based on the 4 mm cutoff [2] and 3 mm cutoff points [30]. For the purpose of estimating prevalence, all inconclusive reactions were classified negative. Prevalence of tuberculin reactors by district and overall prevalence were estimated with 95% confidence intervals using the survey command in STATA. Data were defined by selecting strata (district) and primary sampling units (herds). A variable (initial skin thickness) was generated for each animal based on the average of the initial skinfold thickness at the bovine (Bov0) and avian injection sites (Av0). We used the one-way analysis of variance (ANOVA) to test the equality of the means of initial skin thickness in the three districts based on the assumptions that the variances were the same across districts. Bonferroni, Scheffe, and Sidak multiple comparison tests (in STATA) were used to identify the differences between each pair of means. The relationship between initial skin thickness and tuberculin reactivity was investigated using scatterplots and regression analysis.

#### 2.7.2. Brucellosis

Proportions of positive animals, with 95% confidence intervals were estimated for each district and all the districts (*n* = 395) for the RBT and c-ELISA test results. Proportion estimates were done using the survey command in STATA, with the variables “district” and “herd” set as strata and primary sampling units, respectively. We used c-ELISA results (outcome) in determining brucellosis seroprevalence. The agreement between RBT-1, RBT-2, and c-ELISA test results was investigated using the Kappa agreement test command in STATA. 

## 3. Results 

### 3.1. Tuberculosis

The overall prevalence of BTB based on the 4 mm and 3 mm cut-off criteria was 4.8% (95% CI: 2.6–7.0%) and 6.3% (95% CI: 3.8–8.8%), respectively. TB-reactivity varied according to study area with Monze district recording the highest prevalence (Tables 2 and 4). Changing the cut-off point from 4 mm to 3 mm did not significantly affect the estimated prevalence although on a relative scale the 3 mm detected slightly more positives, as would be expected (Tables 3 and 4).

**Figure 1:**
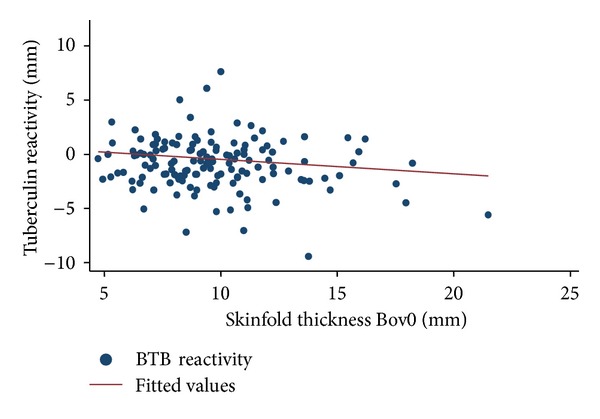
Scatterplot and regression line showing the relationship between BTB reactivity ((Bov72 − Bov0) − (Av72 − Av0)) and intial skinfold thickness at bovine site (Bov0) for all the districts (*n* = 459). BTB reactivity was observed to decrease with increasing skinfold thickness. Skin fold thickness at the bovine site was negatively correlated with tuberculin reactivity.

**Figure 2:**
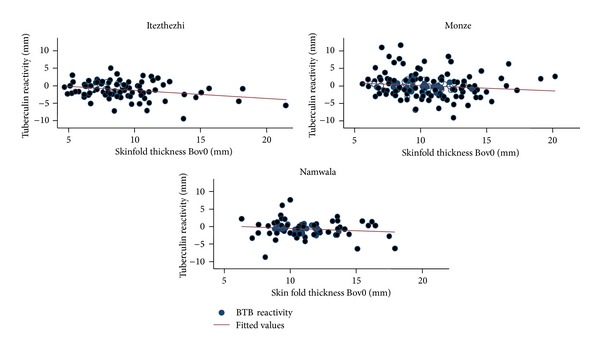
Scatterplot and regression line showing the relationship between BTB reactivity ((Bov72 − Bov0) − (Av72 − Av0)) and intial skinfold thickness at bovine site (Bov0) by District (Itezhitezhi, *n* = 102; Monze, *n* = 176; Namwala, *n* = 181) where reduced BTB reactivity was observed with increasing skinfold thickness. Skin fold thickness at the bovine site was negatively correlateded with tuberculin reactivity.

The mean initial skin thickness was observed to be different between the three districts (*F* (2,377) = 5.1; Prob  *F* ≥ 0.0063). The statistic for Bartlett's test for equal variances (chi2 (2) = 0.823; Prob > chi2 = 0.663) was small, confirming that the assumption of equal variance was not violated in these data, and therefore, the use of ANOVA was a reasonable approach. All the three tests (Bonferroni, Scheffe, and Sidak) showed that there was a significant difference in the means of initial skin thickness between Monze and Itezhitezhi and between Itezhitezhi and Namwala districts (*P* < 0.05), but no difference existed between Monze and Namwala districts (*P* > 0.05). Skinfold thickness at the bovine site (Bov0) was significantly different (*P* < 0.001) from that at the avian site (Av0). The scatter plot and regression line (Figures 1 and 2) showed an association between BTB reactivity ((Bov72 − Bov0) − (Av72 − Av0)) and initial skin fold thickness at the bovine site (Bov0), with thin-skinned animals having a tendency to have increased TB-reactivity.

### 3.2. Brucellosis

A total of 395 animals from Monze (*n* = 176), Namwala (*n* = 118), and Itezhitezhi (*n* = 101) districts of Zambia were tested for brucellosis using RBT and c-ELISA. The overall seroprevalence was 20.7% (95% CI: 17.0–24.4) based on c-ELISA results only. We could not include the RBT results in our final estimation of seroprevalence because there was a significant difference (*P* < 0.001) in RBT test results between the two technicians, who estimated different proportions (Table 5). RBT results from technician (RBT2), with unaided sight, had a better agreement (84.1%) with that of c-ELISA (Table 5). Seroprevalence of brucellosis significantly varied across districts (*P* < 0.001) with Itezhitezhi recording the highest seroprevalence (Table 6). 

## 4. Discussion

We estimated the prevalence of tuberculosis and brucellosis in cattle among traditional cattle in Southern Province of Zambia. We could not perform a random survey because sampling was based on operational areas of the project that funded the study. Despite the above shortcoming, the study gives an indication of the situation regarding the two zoonotic diseases in the study areas. Our estimated overall BTB prevalence of 6.3 (95% CI: 3.8–8.8) is similar to that estimated by Munyeme et al. [34] 6.8% (95% CI: 4.2, 9.5%). This is not surprising considering that the animals investigated were managed under similar husbandry systems and ecological settings as described in the study by Munyeme et al. [34]. Corroboration of the results from the two studies further assures that the study had reasonable external validity despite the limited sample size. When compared to the BTB prevalance recorded in other studies, 3.85% reported in Malawi [30]; 1.3% in Tanzania [33]; 1.4% reported in Uganda [35], this prevalence seems to be slightly high and should raise public health concerns. In a recent national survey in Zambia conducted in humans, the prevalence of BTB in humans was estimated at 0.7% (6/883) (Grace Mbulo, personal communication, 2011). As mentioned earlier, the type of husbandry practice, where majority of cattle herds spent considerable time on the wetlands of Kafue flats and also had regular contact with Kafue lechwe that has high BTB prevalence (27.7% (95% CI: 19.6, 35.9%) [34], could explain the observed difference. 

Considering that the tuberculin test is not a perfect test, some animals could have been missed resulting in underestimation of the prevalence. There are several seasons why tuberculosis-infected animals may give a false negative result. In endemic areas, delayed hypersensitivity may not develop for a period 3–6 weeks following infection, and in chronically infected animals with severe pathology, the tuberculin test may be unresponsive [2]. This situation is likely to be found in endemic areas such as those in our study areas and may lead to an increase in false negatives with subsequent underestimation of prevalence. The possible confounding effect of animal skin thickness on the interpretation of TB-reactivity has been previously described [30]. Our study indicates that TB-reactivity decreased with increasing initial skin thickness among Zebu and Sanga cattle breeds, which is contrary to what has been observed in a study in Malawi [30]. The reason for this is not easily discernable. Despite this contradiction, it is apparent that initial skinfold thickness could confound the interpretation of TB-reactivity. However, due to the limited sample size, no equivocal statement can be made and this need further investigation. 

The RBT is a very sensitive test that sometimes gives a positive result because of S19 vaccination or other cross-reactions such as reaction with *Yersinia enterocolitica* 09 [36]. Sometimes, false-positive serological reactions occur, mostly due to prozoning effect which could be resolved by diluting the serum sample or retesting after 4–6 weeks [2]. Despite this, RBT is recommended as a screening test for detecting infected herds or to guarantee the absence of infection in brucellosis-free herds [2]. The low agreement between RBT-1 and RBT-2 underscores the subjectivity of this assay and the need for quality control in its application. 

The observed *Brucella* seroprevalence in this study is also similar to that earlier reported in traditional cattle 21.6%, (95% CI: 14.2–29.1%) [32]. In this ecosystem, differences in prevalence of both BTB and brucellosis are mostly influenced by type of husbandry practices and contact with the Kafue lechwe on the flood plains of the Kafue River [37, 38]. 

## 5. Conclusion

This study has demonstrated that BTB and brucellosis are a problem in the investigated areas and are likely to pose significant public health risk to traditional farmers. There is a need to control the prevalence of these zoonoses in order to protect the general public considering that the investigated diseases are both milk borne. Further studies need to be conducted to determine the actual public health burden on the affected communities.

## Figures and Tables

**Table 1:** Study areas with estimated livestock populations in the study districts (2006).

Areas (district)	Estimated cattle population	Estimated goat population	Estimated sheep population
Choma	78,521	31,553	2,538
Itezhitezhi	40,250	1,385	128
Monze	110,000	32,340	757
Namwala	99,038	7,600	231

*Information supplied by district veterinary officers from their annual census.

**Table 2:** Study areas with target herds and cattle sample sizes.

Study area (district)	Estimated number of herds (approx. herd size = 100 cattle)	Estimated number of herds to be sampled	Estimated number of animals to be tested (10% sampling fraction)
Choma	785	52	520
Itezhitezhi	402	26	260
Monze	1100	72	720
Namwala	990	65	650

**Table 3:** Distribution of tuberculosis cattle reactors by district at >4 mm cutoff (2008).

District	Total tested	BTB tuberculin reactors (mm)			Avian reactors (4 mm) ((Av_72_ − Av_0_) − (Bov_72_ − Bov_0_))	Average skin thickness Bovine site (Bov_0_) (mm)
		((Bov_72_ − Bov_0_) − (Av_72_ − Av_0_))				
		Negative (<1 mm)	Inconclusive (1–4 mm)	Positive (>4 mm)		
Itezhitezhi	102	81.2 (73.2–89.2)	17.7 (9.8–25.6)	1.0 (0.0–3.1)	15.4 (9.9–20.9)	10.4 (9.6–10.7)
Monze	176	69.3 (62.8–75.8)	22.1 (15.6–28.6)	8.6 (4.1–13.0)	15.3 (8.4–22.2)	10.5 (10.8–11.9)
Namwala	181	84.0 (76.3–91.7)	14.7 (7.2–22.2)	1.3 (0.0–3.9)	38.2 (0.0–85.0)	11.4 (10.8–11.9)
All districts	459	76 (71.4–80.7)	19.2 (14.8–23.6)	4.8 (2.6–7.0)	21.6 (8.5–34.8)	10.8 (10.5–11.1)

**Table 4:** Distribution of tuberculosis cattle reactors by district at ≥3 mm cutoff (2008).

District	Total tested	BTB tuberculin reactors (mm)			Avian reactors (4 mm) ((Av_72_ − Av_0_) − (Bov_72_ − Bov_0_))	Average skin thickness (Bov_0_ + Av_0_/2)
		((Bov_72_ − Bov_0_) − (Av_72_ − Av_0_))				
		Negative (<1 mm) %	Inconclusive (+1–+2 mm) %	Positive (≥3 mm) %		
Itezhitezhi	102	81.2 (73.2–89.2)	16.7 (9.3–24.0)	2.1 (0.0–4.9)	23.1 (14.5–31.6)	10.4 (9.6–10.7)
Monze	176	69.3 (62.8–75 8)	20.2 (13.9–26.6)	10.4 (5.8–15.0)	22.4 (16.3–28.5)	10.5 (10.8–11.9)
Namwala	181	84.0 (76.3–91.7)	13.3 (6.0–20.6)	2.6 (0.0–6.3)	41.8 (0.0–84.0)	11.4 (10.8–11.9)
All districts	459	76.0 (71.4–80.7)	17.7 (13.4–21.9)	6.3 (3.8–8.8)	28.0 (15.7–40.2)	10.8 (10.5–11.1)

**Table 5:** Comparison of RBT results from two technicians and c-ELISA results (*n* = 395).

	Agreement (%)	Expected agreement (%)	Kappa	Std. err.	*P* value
RBT1_RBT2	64.8	54.7	0.22	0.040	0.0000
RBT1_c-ELISA	71.2	54.1	0.37	0.044	0.0000
RBT2_c-ELISA	84.1	69.9	0.47	0.050	0.0000

NB: RBT1: RBT test results from an elderly technician (1) with aided sight; RBT2: RBT test results from a young technician without sight defect correction; Std. err.: standard error.

**Table 6:** Distribution of* Brucella* seropositive cattle (*n* = 395) in Southern Province of Zambia (2008).

District	Total tested	c-ELISA Seroprevalence 95% Confidence interval	RBT-1% (95% Confidence interval)	RBT-2% (95% Confidence interval)
Itezhitezhi	101	33.7 (24.7–42.7)	31.0 (22.3–39.8)	36.3 (27.0–45.6)
Monze	176	19.3 (14.0–24.7)	24.5 (17.5–31.6)	8.18 (2.8–13.6)
Namwala	118	11.9 (6.2–17.5)	60.1 (52.7–67.5)	9.3 (5.5–13.0)
All districts	395	20.7 (17.0–24.4)	42.7 (38.1–47.3)	15.9 (12.6–19.3)
